# Identifying the Sources of Intestinal Colonization With Extended-Spectrum β-Lactamase-Producing *Escherichia coli* in Healthy Infants in the Community

**DOI:** 10.3389/fmicb.2022.803043

**Published:** 2022-03-31

**Authors:** Mohammed Badrul Amin, Kazi Injamamul Hoque, Subarna Roy, Sumita Rani Saha, Md. Rayhanul Islam, Timothy R. Julian, Mohammad Aminul Islam

**Affiliations:** ^1^Laboratory of Food Safety and One Health, Laboratory Sciences and Services Division, International Centre for Diarrhoeal Disease Research, Bangladesh (icddr, b), Dhaka, Bangladesh; ^2^Eawag, Swiss Federal Institute of Aquatic Science and Technology, Dübendorf, Switzerland; ^3^Swiss Tropical and Public Health Institute, Basel, Switzerland; ^4^University of Basel, Basel, Switzerland; ^5^Paul G. Allen School for Global Health, Washington State University, Pullman, DC, United States

**Keywords:** antibiotic resistance, transmission, colonization, whole genome sequencing, *E. coli*, ESBL, children, drinking water

## Abstract

The prevalence of fecal colonization with extended-spectrum β-lactamase-producing *Escherichia coli* (ESBL-Ec) among children in low- and middle-income countries is alarmingly high. This study aimed to identify the sources of ESBL-Ec colonization in children < 1 year old through comparative analysis of *E. coli* isolates from child stool, child’s mother stool, and point-of-use drinking water from 46 rural households in Bangladesh. The pairwise similarity in antibiotic susceptibility of *E. coli* from all three sources was evaluated, followed by phylogenetic clustering using enterobacterial repetitive intergenic consensus polymerase chain reaction and whole-genome sequence analysis of the isolates. Matching antibiotic susceptibility and enterobacterial repetitive intergenic consensus polymerase chain reaction patterns were found among ESBL-Ec isolates from child–mother dyads of 24 and 11 households, respectively, from child–water dyads of 5 and 4 households, respectively, and from child–mother–water triads of 3 and 4 households, respectively. Whole-genome sequence analysis of 30 isolates from 10 households revealed that ESBL-Ec from children in five households (50%) was clonally related to ESBL-Ec either from their mothers (2 households), drinking water sources (2 households), or both mother and drinking-water sources (1 household) based on serotype, phylogroup, sequence type, antibiotic resistance genes, mobile genetic elements, core single-nucleotide polymorphisms, and whole-genome multilocus sequence typing. Overall, this study provides empirical evidence that ESBL-Ec colonization in children is linked to the colonization status of mothers and exposure to the household environments contaminated with ESBL-Ec. Interventions such as improved hygiene practices and a safe drinking water supply may help reduce the transmission of ESBL-Ec at the household level.

## Introduction

Antimicrobial resistance is a growing public health challenge expected to disproportionately affect low- and middle-income countries in the coming decades (Nadimpalli et al., 2021). The rate of intestinal colonization with antibiotic-resistant organisms has been increasing globally, especially in low- and middle-income countries where both exposure to antibiotic-resistant organisms and use of antibiotics are high (Woerther et al., 2013). Children younger than 5 years, including infants, are frequent carriers of antibiotic-resistant bacteria and antimicrobial resistance genes (ARGs) in their gut (Kaarme et al., 2013; Fernandez-Reyes et al., 2014; Farra et al., 2016; Islam et al., 2018). In rural Bangladesh, we previously found that around 78% of healthy infants (asymptomatic) younger than 1 year were colonized by extended-spectrum β-lactamase-producing *Escherichia coli* (ESBL-Ec) with a mean of 6.21 log_10_ CFU/g wet weight of stool (Islam et al., 2019). Moreover, ESBL-Ec encompassed an average of one-third of the total culturable *E. coli* found in stool samples. Intestinal colonization with ESBL-Ec is associated with an increased risk of drug-resistant infections, treatment complications, and mortality (Ben-Ami et al., 2009; Freeman et al., 2012; Van Aken et al., 2014; Rottier et al., 2015; Isendahl et al., 2019), as it has been shown that multidrug-resistant (MDR) commensal *E. coli* causes extraintestinal infections such as urinary tract infection, bloodstream infections, and neonatal meningitis (Poolman and Wacker, 2016). Additionally, gut colonization with ESBL-Ec represents a gene pool for ARGs that can be exchanged with human pathogens in the gut (Gekenidis et al., 2020).

*Escherichia coli* is one of the most common commensal gut microflora that account for a significant proportion of antibiotic-resistant bacteria both in infants and adults (Moore et al., 2013; Backhed et al., 2015; Pärnänen et al., 2018). *E. coli* is also one of the first bacterial species to colonize an infant’s gut through exposure to maternal fecal flora and/or environmental bacteria (Ducluzeau, 1993; Nowrouzian et al., 2003; Hetzer et al., 2019). *E. coli* also share antibiotic resistance determinants with other pathogenic and non-pathogenic organisms *via* horizontal gene transfer of mobile genetic elements (MGEs), including plasmids, integrons, or transposons (Wright, 2007). Therefore, reducing the level of gut colonization with antibiotic-resistant organisms is considered an effective strategy to control infections caused by MDR organisms in hospitals and in the community (Karanika et al., 2016). Identifying the sources and transmission routes of resistant organisms that colonize infants’ gut will be helpful for designing effective intervention strategies.

The antibiotic resistome (collection of antibiotic resistance genes) and mobilome (collection of MGEs) present in a child’s gut are influenced by diet and the external environment (Wu et al., 2016; Li et al., 2021). For example, maternal gut microbiome and breast milk microflora influence child microbiomes (Pärnänen et al., 2018). The gut microbiome of children in resource-poor settings is also impacted by environmental flora, as children are frequently exposed to contaminated environments such as courtyard soil, toys and other inanimate objects, contaminated complementary foods, and drinking water (Hartinger et al., 2021). In low-income settings, complementary foods are often contaminated with microorganisms that can cause diseases in infants, and unhygienic food preparation practices are potential sources of contamination (Islam et al., 2012).

In both urban and rural Bangladesh, point-of-use drinking water is found to be frequently contaminated with fecal indicator bacteria that are resistant to antibiotics (Hoque et al., 2006; Talukdar et al., 2013). Given the frequent contamination of point-of-use drinking water within households, as demonstrated by higher levels of contamination compared with water at the source, drinking water may be an important route of exposure to antibiotic-resistant bacteria (Ercumen et al., 2015). However, empirical evidence demonstrating household stored water as a reservoir of antibiotic-resistant bacteria is scarce.

In this study, we analyzed *E. coli* isolates from mother–child dyads along with stored drinking water in a number of rural households in Bangladesh. The findings of the study provide important insights into the possible intra-household transmission of ESBL-Ec from mother and/or household drinking water to children.

## Materials and Methods

### Study Participants Enrollment and Sample Collection

A total of 100 households having an infant (≤ 1 year) were randomly selected during the period from March to October 2017 in rural villages of Hajigonj (*n* = 50) and Matlab (*n* = 50), two subdistricts of Chandpur, Bangladesh. Infant’s mothers were enrolled in this study upon written consent either by signature or by thumbprint if they were not literate and were approached for stool sample collection in a sterile container provided by our field staff. Infant demographic information, including age, mode of delivery, feeding practices, and antibiotic consumption within 3 months before sampling, was collected (Supplementary Table 1). Three types of samples, including mother stool (MS), child stool (CS), and point-of-use drinking water (WU), were collected on the same day from each household and marked as Hajigonj and Matlab sample ID as RH and RM, respectively. All samples were immediately kept in a cold box (4–8°C) and transported to the laboratory within 4–6 h.

### Detection and Isolation of *Escherichia coli*

*Escherichia coli* was isolated from stool and water samples following a standard culture-based procedure described previously (Islam et al., 2019). Briefly, 50 μl of each of four 10-fold serial dilutions (10^–1^ to 10^–4^) of each stool sample from mothers and children was inoculated using drop plate techniques onto MacConkey agar plates (Becton Dickson, MD, United States) and then incubated at 37°C for 18 h. For water samples, 100 ml was filtered through a 0.45-μm membrane filter, which was placed on modified membrane thermotolerant *E. coli* agar (m-TEC) (Becton Dickinson, MD, United States) according to the procedure described by the US Environmental Protection Agency (Method 1603, EPA) (US Environmental Protection Agency, 2002). At least one well-isolated colony typical of *E. coli* from each MacConkey and m-TEC plate was tested by API-20E (bioMerieux, France) for confirmation and stored at −80°C.

### Antibiotics Susceptibility Test

Standard disk diffusion technique following the Clinical and Laboratory Standards Institute guidelines was used to determine antibiotic susceptibility (AS) of *E. coli* (Patel, 2017). The list of antibiotics used in this study included ampicillin (10 μg), gentamycin (10 μg), tetracycline (30 μg), meropenem (10 μg), imipenem (10 μg), ceftriaxone (30 μg), cefotaxime (30 μg), cefepime (30 μg), ciprofloxacin (5 μg), nalidixic acid (30 μg), azithromycin (15 μg), trimethoprim/sulfamethoxazole (25 μg), and chloramphenicol (30 μg) (Oxoid, Hampshire, United Kingdom). The isolates were classified as resistant or sensitive by measuring the zone of inhibition according to the Clinical and Laboratory Standards Institute (Patel, 2017). If an isolate was resistant to at least one agent in three or more classes of antibiotics, it was categorized as MDR (Magiorakos et al., 2012). Third-generation cephalosporin-resistant *E. coli* isolates were further tested for ESBL using the combined disk test described previously (Patel, 2017).

### Typing of *Escherichia coli* Isolates by Enterobacterial Repetitive Intergenic Consensus Polymerase Chain Reaction

All *E. coli* isolates were analyzed using enterobacterial repetitive intergenic consensus polymerase chain reaction (ERIC-PCR) according to the procedure described earlier (Morales-Erasto et al., 2011). ERIC-PCR was carried out in a Thermal Cycler system (C1000 Touch, BioRad, Hercules, CA, United States), and amplified products were separated in 1.5% agarose gel, normalized using the 100-bp DNA ladder as an external reference standard, stained with Midori Green, and visualized by FastGene Blue/Green LED Gel Illuminator (Nippon Genetics, Tokyo, Japan). The TIF formatted image was analyzed with BioNumerics version 4.5 (Applied Maths, Kortrijk, Belgium) to determine the similarity among the isolates. A dendrogram showing the degree of similarity among isolates was generated by Dice, and clustering correlation coefficients were calculated by the unweighted pair group method with arithmetic averages. In ERIC-PCR typing, clusters were considered at approximately 85% similarity in banding patterns between *E. coli* isolates (Casarez et al., 2007).

### DNA Extraction and Whole-Genome Sequencing of *Escherichia coli* Isolates

DNA was extracted from an overnight culture of *E. coli* isolates using the Maxwell culture DNA extraction kit and Maxwell automated nucleic acid extraction system (Promega, Madison, USA) following the manufacturer’s instruction. The purity and concentration of the extracted DNA were evaluated using NanoDrop spectrophotometer (Thermo Scientific, United States) and Qubit 2.0 fluorometer (Life Technologies, Carlsbad, CA, United States), respectively. Libraries were prepared using the Nextera XT kit, and paired-end sequencing was performed using the Illumina NextSeq500 platform (Illumina, San Diego, CA, United States). Whole-genome sequencing (WGS) data of 30 *E. coli* isolates from 10 households were submitted to the National Center for Biotechnology Information under the accession numbers listed in Supplementary Table 2. The size of the assembled genome of *E. coli* isolates ranged from 4.39 to 5.43 Mb with a mean guanine–cytosine (GC) content of 49.6%.

### Bioinformatics Analyses

FastQC version 0.11.4 was used for assessing the quality of the reads^1^. Reads were *de novo* assembled using ABySS assembler version 2.2.3 (Simpson et al., 2009). Contigs generated by ABySS were ordered and orientated using ABACAS (Assefa et al., 2009). Prokka 1.12 was used for genome annotation (Seemann, 2014). ParSNP was used for identification of core genome single-nucleotide polymorphisms (SNPs) by comparing to the reference genome of strain *E. coli* SEC470, which showed closed similarity with our study isolates, and construction of phylogenetic tree was done based on aligned core-SNPs (Treangen et al., 2014; Liu et al., 2016). Furthermore, SNP difference was assessed using snp-dists available at https://github.com/tseemann/snp-dists. The sequence types (STs) and serotypes of the isolates were determined using the Achtman scheme and SRST2 v0.2.0^2^, respectively (Inouye et al., 2014). ARGs were identified using ARIBA available at https://github.com/sanger-pathogens/ariba considering identity and coverage threshold greater than 90% against the Comprehensive Antibiotic Resistance Database (Hunt et al., 2017). The presence of virulence genes associated with intestinal pathogenic *E. coli* and plasmid replicons was identified using VirulenceFinder and PlasmidFinder database (Carattoli et al., 2014; Joensen et al., 2014). ClermonTyping was used for *E. coli* phylo-grouping (Beghain et al., 2018). For MGEs, annotation of transposons was done using ISsaga, and integrons were identified using the database reported earlier (Varani et al., 2011; Cury et al., 2016). Whole-genome multilocus sequence typing (wgMLST) of isolates was carried out using Enterobase schemes with 25,002 loci for *E. coli* and minimum spanning tree based on wgMLST generated using MSTree Algorithm (Zhou et al., 2020).

### Statistical Analysis

Data were entered in SPSS 20.0 (IBM Inc., Chicago, IL, United Ststes). Data cleaning statistical analysis was done in R-3.4.2 (R Core Team, 2013). Data visualization was done using MS Office Excel 2010 (Microsoft, Washington, United States). Jaccard/Tanimoto similarity test was done for similarity index analysis among *E. coli* strains isolated from CS, MS, and WU sample types (Chung et al., 2019). AS patterns of isolates were analyzed for similarity using the Jaccard index (J-index), where J > 0.6 is considered a statistically significant association (Kain et al., 2016; Lavers and Bond, 2016).

## Results

### Antibiotic Susceptibility Profiles of *Escherichia coli* From Child Stool, Mother Stool, and Point-of-Use Drinking Water Samples

Of 100 households enrolled in this study, *E. coli* was detected in samples from all three sources (MS, CS, and WU samples) of 55 households. AS tests of *E. coli* from these 55 households showed that all *E. coli* isolates from MS samples were resistant to ampicillin, followed by 98% (*n* = 54) to third-generation cephalosporins (3GC), 55% (*n* = 30) to azithromycin, 53% (n = 29) to fourth-generation cephalosporins (4GC), 31% (*n* = 17) to sulfamethoxazole–trimethoprim, 29% (*n* = 16) to tetracycline, and 27% (*n* = 15) to fluoroquinolones. *E. coli* isolates from CS samples were all resistant to ampicillin, followed by 95% (*n* = 52) to 3GC, 85% (*n* = 47) to azithromycin, 71% (*n* = 39) to 4GC, 49% (*n* = 27) to fluoroquinolones, 40% (*n* = 22) to sulfamethoxazole–trimethoprim, and 27% (*n* = 15) to tetracycline. For *E. coli* from WU samples, 55% (*n* = 30) isolates were resistant to ampicillin, followed by 42% (*n* = 23) to 3GC, 35% (*n* = 19) to sulfamethoxazole–trimethoprim, 33% (*n* = 18) to fluoroquinolones, and 24% (*n* = 13) to 4GC. The prevalence of MDR *E. coli* was high (98%) among isolates from CS samples, followed by MS (87%) and WU (55%). Of 55 households, CS and MS samples from 46 households (designated as HH1 to HH46) were positive for ESBL-Ec, of which 23 households had ESBL-Ec in WU sample.

**FIGURE 1:**
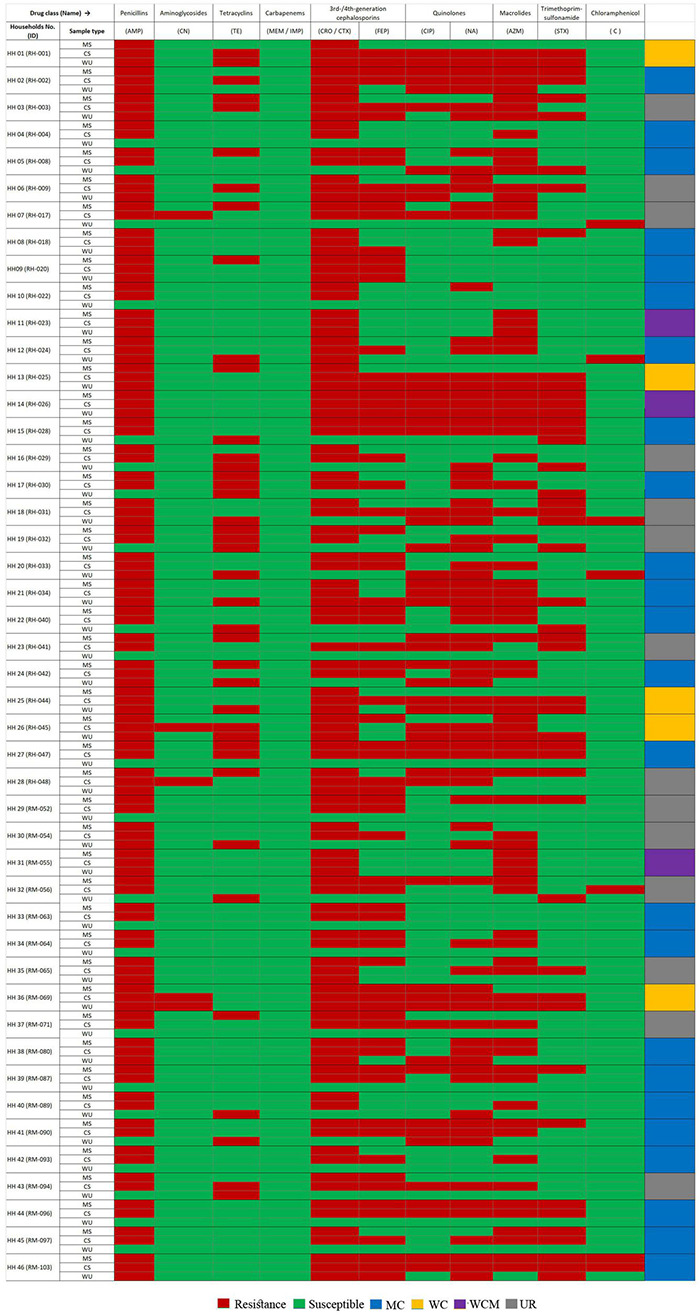
Heatmap showing antibiotic susceptibility (AS) pattern of ESBL-Ec isolated from stool samples of mother–child dyads and point-of-use drinking water samples from 46 households. Amp, ampicillin; CN, gentamycin; TE, tetracycline; MEM, meropenem; IMP, imipenem; CRO, ceftriaxone; CTX, cefotaxime, FEP, cefepime; CIP, ciprofloxacin; NA, nalidixic acid; AZM, azithromycin; STX, trimethoprim/sulfamethoxazole; C, chloramphenicol; HH, households; MS, mother stool; CS, child stool; WCM, AS patterns matched among *E. coli* isolates from mother–child gut and point-of-use drinking water origin; MC, AS pattern matched between *E. coli* isolates of mother–child gut origin; WC; AS pattern matched between *E. coli* strains from child-gut and point-of-use drinking water origin; UR; AS pattern among *E. coli* strains from all sample sources were diverse.

Among these 46 households, *E. coli* isolates from CS and MS samples in 24 households (52%) had matching AS profiles, whereas similar results were observed between CS and WU isolates only in five households (11%) based on the J-index similarity matrix (Supplementary Table 3). Furthermore, *E. coli* from all three sources (CS, MS, and WU) with matching AS profiles was found only in three households (6%) (Figure 1).

### Enterobacterial Repetitive Intergenic Consensus Polymerase Chain Reaction Patterns of *E. coli* Isolates From Child Stool, Mother Stool, and Point-of-Use Drinking Water Samples

**FIGURE 2:**
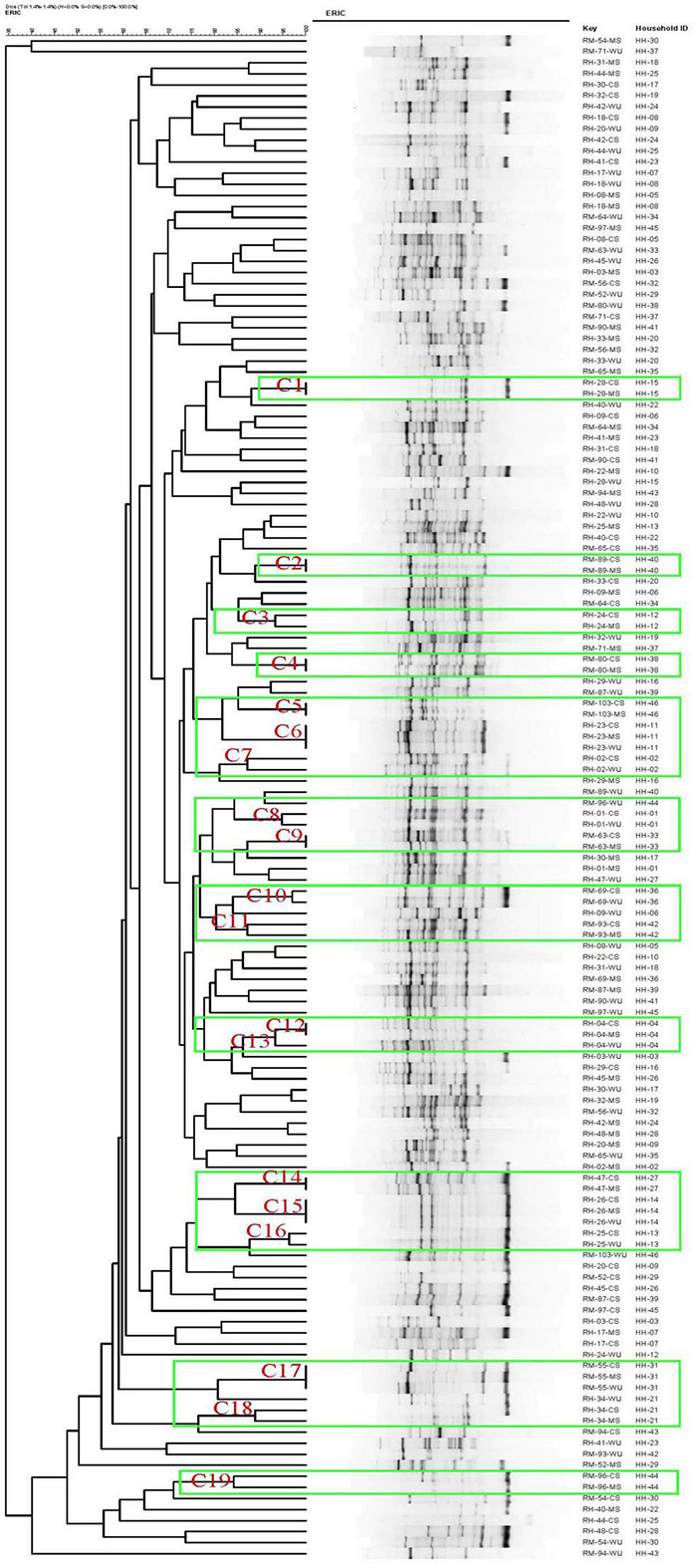
Dendrogram generated by BioNumerics software, showing distances calculated by Dice similarity index of enterobacterial repetitive intergenic consensus polymerase chain reaction banding patterns of *E. coli* strains obtained from stool samples of mother and child and point-of-use drinking water samples from 46 households. Degree of similarity (%) is shown on scale.

Considering a similarity cutoff of 85% among ERIC-PCR banding patterns, a total of 138 isolates consisting of 92 from CS and MS (46 each) and 46 from WU (23 each of ESBL-Ec and non-ESBL-Ec) were grouped into 19 major clusters designated as C1–C19 (Figure 2). CS isolates from 11 (24%) and 4 (9%) households had matching ERIC-PCR patterns with their corresponding MS and WU isolates, respectively. Furthermore, isolates from all three sources (MS, CS, and WU) from four additional households had identical ERIC-PCR profiles. Overall, ESBL-Ec from children of 19 households (41%) were related to isolates either from their mothers or drinking water or both, whereas isolates from the remaining 27 households (59%) were unrelated to each other (Figure 2).

### Genomic Analysis of *Escherichia coli* Isolates From Child Stool, Mother Stool, and Point-of-Use Drinking Water Samples

Based on ERIC-PCR clustering, we selected 30 isolates from 10 households for WGS analysis. Of these, four households had matching CS-MS isolates (HH4, HH12, HH44, and HH46), two had matching CS–WU isolates (HH1 and HH36), one had matching CS–MS–WU isolates (HH31), and three had unmatched isolates (HH26, HH28, and HH35). Of these 30 isolates, 28 were ESBL-Ec, and the remaining two were non-ESBL-Ec.

WGS analysis revealed that *E. coli* isolates from CS–MS dyads in four households (HH4, HH12, HH44, and HH46) had similar serotype, ST, phylogroup, and pathotype except for HH4 and HH44, where isolates from CS–MS dyads differ only in serotype and phylogroup, respectively. Similarly, *E. coli* from WU–CS dyads from two households (HH1 and HH36) and CS–MS–WU triad from one household (HH31) were identical based on serotype, ST, phylogroup, and pathotype (Table 1).

**TABLE 1:** Distribution of serotype (O and H types), sequence type (ST), phylogroup, and pathotype in 30 *E. coli* isolates obtained from 10 households.

HH^a^ No.	HH ID	H type	O type	ST^b^ type	Phylogroup^c^	Pathotype^d^
HH-01	RH-01-MS	H21	O28ac-O42	Unknown	B1	-
	RH-01-CS	H9	Onovel14	410	C	-
	RH-01-WU	H9	Onovel14	410	C	-
HH-04	RH-04-MS	H27	O51	226	A	-
	RH-04-CS	H10	Onovel17	226	A	-
	RH-04-WU	H42	O157	3,744	A	astA(STEC)
HH-12	RH-24-MS	H33	O99	10	A	aaiC (EAEC), astA(STEC)
	RH-24-CS	H33	O99	10	A	aaiC (EAEC), astA(STEC)
	RH-24-WU	H21	Onovel4	602	B1	
HH-26	RH-45-MS	H10	O45	226	A	astA(STEC)
	RH-45-CS	H30	O153	315	D	-
	RH-45-WU	H9	Onovel32	10	A	-
HH-28	RH-48-MS	H23	O8	224	B1	-
	RH-48-CS	H4	O25	131	B2	aatA(EAEC)
	RH-48-WU	H41	O137	3,018	E	
HH-31	RM-55-MS	H18	O17-O44-O77	394	D	aatA(EAEC), astA(STEC)
	RM-55-CS	H18	O17-O44-O77	394	D	aatA(EAEC), astA(STEC)
	RM-55-WU	H18	O17-O44-O77	394	D	aatA(EAEC), astA(STEC)
HH-35	RM-65-MS	H48	O164	2,705	A	-
	RM-65-CS	H15	O23	70	D	astA(STEC)
	RM-65-WU	H12	O8	3,580	B1	-
HH-36	RM-69-MS	H21	not found	443	B1	-
	RM-69-CS	H4	O25	131	B2	-
	RM-69-WU	H4	O25	131	B2	-
HH-44	RM-96-MS	H30	O153	38	D	aatA(EAEC)
	RM-96-CS	H30	O153	38	G	aatA(EAEC)
	RM-96-WU	H16	O185	2,280	B1	-
HH-46	RM-103-MS	H19	Onovel1	1,290	A	-
	RM-103-CS	H19	Onovel1	1,290	A	-
	RM-103-WU	H18	O17-O44-O77	8,131	G	aatA(EAEC), astA(STEC)

*^a^Household number.*

*^b^Sequence type (ST) based on multilocus sequence typing Achtman scheme (Achtman et al., 2012).*

*^c^Phylogenetic group based on Clermon Typing (Beghain et al., 2018).*

*^d^Pathotype: EPEC: eae, bfp, and perA; EAEC: aatA; EIEC: ipaH and ial; ETEC: eltA, eltB, and lt; EHEC: espK, espN, nleA, nlec, and nleG; STEC: astA, aaic, stx1a, stx1b, stx2a, and stx2db (Joensen et al., 2014).*

**FIGURE 3:**
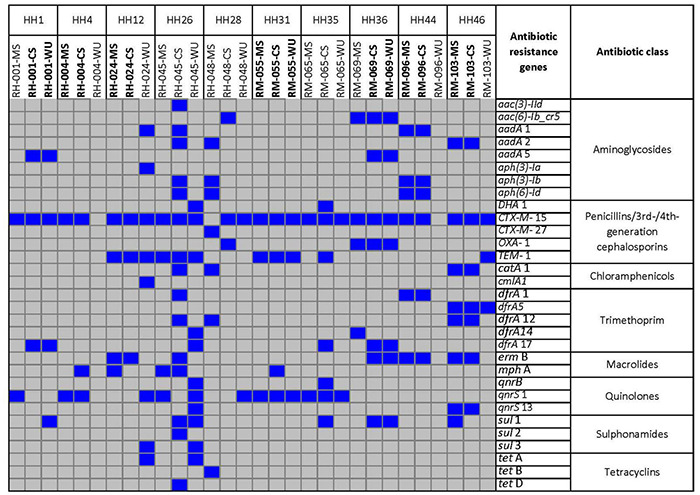
Distribution of antibiotic resistance genes retrieved from whole-genome sequence of 30 *E. coli* strains isolated from CS, MS, and WU samples from 10 households. Blue and gray boxes indicate presence and absence of genes, respectively. HH, households; MS, mother stool; CS, child stool; WU, point-of-use drinking water.

A total of 31 different antibiotic resistance genes were found among these *E. coli* isolates (Figure 3). Antibiotic resistance genes that were commonly found in both CS and MS isolates were as follows: *bla*_CTX–M–15_ (β-lactum) and *qnrS*1 (quinolones) in HH4; *bla*_CTX–M–15_, *bla*_TEM–1_ (β-lactum), and *ermB* (macrolide) in HH12; *aadA*1, *aph(3)-Ib*, *aph(6)-Id* (aminoglycosides), *bla*_CTX–M–15_, *dfrA*1 (trimethoprim), and *ermB* in HH44; and *aadA*2, *bla*_CTX–M–15_, *catA*1 (chloramphenicol), *dfrA*5, *dfrA*12, *ermB*, and *qnrS*13 for HH46. The common genes among CS–WU isolates from HH1 were *aadA*1, *bla*_CTX–M–15_, and *dfrA*17, whereas *aac(6)-Ib-cr*5 (aminoglycoside), *bla*_CTX–M–15_, *bla*_OXA–1_, *dfrA*17, *ermB*, and *sul*1 (sulphonamides) were common in HH36. Furthermore, *bla*_CTX–M–15_, *bla*_TEM–1_, and *qnrS*1 genes were commonly found in ESBL-Ec from CS–MS–WU isolates in HH31.

Same MGEs were found among isolates from multiple sources within the same households. Plasmids that belonged to the IncF family (FIA, FIB, FIC, and FII) along with the class I integron (*Int*1) and Tn3 transposon were most commonly found in isolates from different households (Table 2). In one household (HH46), IncB/O/K/Z plasmid was detected in both CS and MS isolates.

**TABLE 2:** Distribution of mobile genetic elements in 30 *E. coli* isolates of 10 households.

HH ID	*E. coli* isolates ID	Plasmid Inc^a^	Integron	Transposon
HH-01	RH-001-MS	N/F	-	Tn3(R)
	RH-001-CS	IncFIA, IncFIB, IncFII	*Int* 1, *int* 2	Tn3(CR)
	RH-001-WU	IncFIA, IncFIB, IncFII	*Int* 1, *int* 2	Tn3 (CR)
HH-04	RH-004-MS	N/F	-	Tn3(R)
	RH-004-CS	N/F	-	Tn3(R)
	RH-004-WU	N/F	-	-
HH-12	RH-024-MS	IncFII, IncI1, IncX4	-	Tn3(R)
	RH-024-CS	IncFII, IncI1, IncX4	-	Tn3(R)
	RH-024-WU	IncFIB, IncFIC, IncX1	*Int* 1	Tn3(R)
HH-26	RH-045-MS	IncFIA, IncFII	-	Tn3(R)
	RH-045-CS	IncFIB, IncFII	*Int* 1, *int* 2	Tn3(R)
	RH-045-WU	IncFIB, IncFII, IncX1	Int 1	Tn3(R)
HH-28	RH-048-MS	IncFIA, IncFIB, IncFII	*Int* 1	-
	RH-048-CS	IncFIA, IncFIB, IncFII	*Int* 1	-
	RH-048-WU	IncX4	-	Tn3(R)
HH-31	RM-055-MS	IncFIB, IncFII	-	Tn3(R)
	RM-055-CS	IncFIB, IncFII	-	Tn3(R)
	RM-055-WU	IncFIB, IncFII	-	Tn3(R)
HH-35	RM-065-MS	N/F	-	Tn3(R)
	RM-065-CS	IncB/O/K/Z, IncFII	*Int* 1	Tn3(R)
	RM-065-WU	N/F	-	Tn3(R)
HH-36	RM-069-MS	IncFIA, IncFIB, IncFII	*Int* 1	Tn3(R)
	RM-069-CS	IncFIA, IncFII	*Int* 1, *int* 2, *Int*3	Tn3(CR)
	RM-069-WU	IncFIA, IncFII	*Int* 1, *int* 2, *Int*3	Tn3(R)
HH-44	RM-096-MS	IncFIB, IncFII	*Int* 1	Tn3(R)
	RM-096-CS	IncFIB, IncFII	*Int* 1	Tn3(R)
	RM-096-WU	N/F	-	Tn3(R)
HH-46	RM-103-MS	IncB/O/K/Z	*Int* 1	Tn3(R)
	RM-103-CS	IncB/O/K/Z	*Int* 1	Tn3(R)
	RM-103-WU	IncB/O/K/Z, IncFIB, IncFII	-	-

*^a^Plasmid Inc type based on Carottoli (Carattoli et al., 2014).*

**FIGURE 4:**
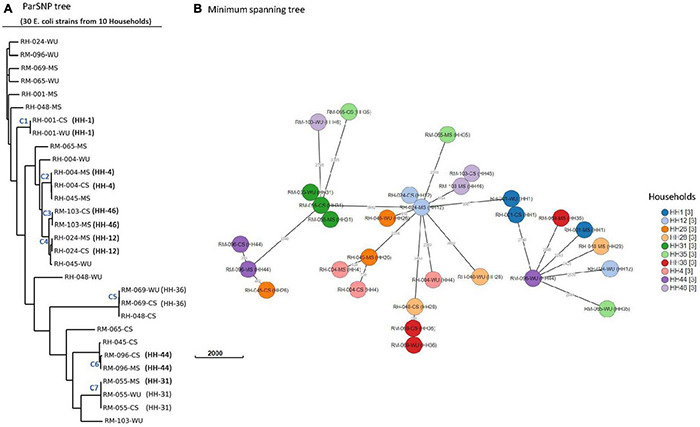
Phylogenetic analysis of 30 *E. coli* isolates. **(A)** Core-genome SNP tree using ParSNP alignment. **(B)** Minimum spanning tree generated by wgMLST. Isolates from same household are shown by circles of identical color. Numbers next to branches indicate allelic differences according to wgMLST analysis.

Genetic relatedness among *E. coli* isolates was determined using core genome SNPs and wgMLST (Figure 4). Core SNPs analysis of the isolates produced seven clusters (C1–C7), each representing one household (Figure 4A). CS isolates from 4 (HH04, HH12, HH44, and HH46), 2 (HH01 and HH36), and 1 (HH31) households were clustered together with isolates from the corresponding MS, WU, and both, respectively. Pairwise core SNP difference among isolates in clusters C1 and C3–7 was minimum (less than 21 SNPs) and monophyletic (share most common recent ancestor and include all its descendants) in phylogenetic analysis, suggesting clonality among the isolates. The SNP difference among isolates in cluster C2 was relatively higher (1,231 SNPs), and they had a paraphyletic (share a common ancestor, but some descendants are excluded) relationship, suggesting that these isolates are distantly related. The core SNP difference among isolates in the remaining three households (HH26, HH28, and HH35) was very high (> 18,000), suggesting that they are unrelated to each other (Supplementary Table 4). A minimum spanning tree was also generated based on wgMLST to further assess the genetic relatedness (Figure 4B). wgMLST result shows that allelic distances among CS isolates in five households were < 5 loci from their corresponding MS (HH12 and HH46) or WU (HH1 and HH36) or both (HH31) isolates, indicating their clonal relationship.

## Discussion

In this study, we investigated genomic characteristics of *E. coli* isolates from MS, CS, and WU samples from the same households in rural Bangladesh to identify the potential sources of ESBL-Ec colonized in the intestine of children (Islam et al., 2019). We found concordant results between ERIC-PCR typing and WGS based analysis of isolates.

WGS analysis has increasingly been used as an essential tool for microbial source tracking (Rantsiou et al., 2018). The previous report suggests that isolates are predicted to be clonal or originated from the same ancestral clone when pairwise SNP difference is < 21, the allelic difference in wgMLST is 0–17 loci, and monophyletic topology is present in the phylogenetic tree (Pightling et al., 2018; Blanc et al., 2020). The report also added that a more precise decision on the clonality of isolates could be made from WGS-based analysis when it is interpreted along with available epidemiological data. In this study, 15 (33%) of 46 households had identical ERIC-PCR banding patterns of ESBL-Ec isolates from mother–child dyads, indicating possible transmission of organisms from one source to the other within the same household. The result is also supported by WGS analysis, where three households (HH12, HH31, and HH46) had identical ESBL-Ec strains in mother and child pairs based on the core-SNPs and wgMLST analysis. Clonal relationship between isolates from CS–MS dyads in the same households supports vertical transmission, although the cross-sectional design of the study did not permit us to determine the directionality of transmission. This finding is concordant with the prior work, which showed that there is a significant impact of mother’s gut microflora on the colonization with the antibiotic-resistant microbial community in infant’s gut (Zhang et al., 2011; Denkel et al., 2014; Pärnänen et al., 2018).

In 8 (35%) of 23 households, CS *E. coli* isolates had ERIC-PCR patterns identical to isolates from WU samples, indicating that drinking water could be a potential source of gut colonization with these organisms (Günther and Schipper, 2013). The observation is also supported by WGS analysis where ESBL-Ec from WU samples were found similar with ESBL-Ec isolates from CS samples in three representative households (HH01, HH31, and HH36) based on core-SNP, wgMLST, serotype distribution, phylogroup, antibiotic resistance genes, and MGE characteristics.

MGEs such as plasmids, integrons, and transposons play an important role in the transmission of antibiotic resistance in the bacterial community. In this study, the majority of ESBL-Ec isolates possessed IncF type plasmids, which are commonly found in *E. coli* from different human specimens (e.g., rectal samples, gastric aspirate samples, and vaginal samples) and animal sources (e.g., poultry feces) (Marcadé et al., 2008). Furthermore, the IncF plasmid family is predominantly associated with the transfer of antibiotic resistance traits (Johnson et al., 2007; Lyimo et al., 2016) and can persist under selective environments because of the addiction systems harbored by these plasmids (Yang et al., 2015). Apart from plasmids, *E. coli* isolates possessed mostly *int*1, which is mainly involved in the spread of antibiotic resistance (Domingues et al., 2012). In the present study, all the isolates have *int*1 except one harbored *aadA* encoding aminoglycosides and *dfr*A encoding trimethoprim resistance, which is concordant with the previous studies (Lee et al., 2006; Vinué et al., 2008; Bailey et al., 2010). Transposon Tn3 family and Tn3-like subfamilies play an important role in the dissemination of antibiotic resistance genes, especially the β-lactam and aminoglycoside resistance genes (Tran van Nhieu and Collatz, 1987; Tolmasky, 2000; Partridge and Hall, 2005).

Analysis of WGS data also revealed that 8 of 10 households had at least one *E. coli* positive for pathogenic genes, either *aat*A or *ast*A or both. *aat*A encodes for an autotransporter predominantly found in avian pathogenic *E. coli* and enteroaggregative *E. coli* (EAEC), causing diarrhea in humans. *ast*A is found in multiple pathotypes, including EAEC, enterohemorrhagic *E. coli*, and enteropathogenic *E. coli*. EAEC has been shown to cause subclinical infection, is associated with growth decrements in children in Bangladesh, and plays a significant role as a co-pathogen (Lima et al., 2018). Moreover, *aat*A- and *ast*A-carrying *E. coli* are also prevalent in household environments, including courtyard soil that is commonly impacted by feces of domestic poultry carrying avian pathogenic *E. coli* isolates (Montealegre et al., 2020). Children have direct exposure to soil, as reported by a previous study that more than 80% of children < 30 months of age in rural Bangladesh were observed mouthing soil, objects with visible soil, or food with visible soil (Morita et al., 2017). It is also likely that children < 1 year of age can get the ESBL-Ec present in courtyard soil through their mothers, as women in rural areas are regularly exposed to soil (Montealegre et al., 2018).

Our findings should be interpreted in the context of this study’s limitations. First, only one colony/isolate per sample was analyzed, which is not sufficient to describe the genetic heterogeneity among *E. coli* strains present in a sample. The selection of colonies with different morphological characteristics from each sample would have given us more confidence in drawing the conclusion on the intra-household transmission of ESBL-Ec, which has been underrepresented in this study. Second, only MS and WU samples were included for comparative analysis, and sampling of other household environments such as soil and domestic animals was not done, which might be the potential sources of antibiotic resistance acquisition. Nevertheless, this study was the first attempt to identify potential sources of colonization with ESBL-Ec among children in Bangladesh. Intestinal colonization with ESBL-Ec among children is linked to the colonization status of mothers and exposure to the contaminated household environments, which are exacerbated by the lack of improved sanitation, safe drinking water supply, and hygienic practices. Further studies could be designed to longitudinally follow up the intestinal colonization with ESBL-Ec in children, including other members of the household, to understand the persistence and dynamics of colonization by these organisms in this setting.

## Data Availability Statement

All relevant data presented in this study are within the manuscript. In addition, the draft genome sequences of the 30 *E. coli* isolates are available in GenBank under accession numbers CP050193-CP050222. All the samples are part of BioProject PRJNA607650 and correspond to BioSample IDs SAMN14342432-SAMN14342461 (Supplementary Table 2).

## Ethics Statement

The study (PR-16086) involving human participants was reviewed and approved by the research and the ethical review committees of icddr, b. The review committees also monitored the progress of this study. Written informed consent to participate in this study was provided by the participants’ legal guardian/next of kin.

## Author Contributions

MBA and MAI contributed to the conceptualization and initial study design. MBA wrote the first draft and was involved in methodology and data analysis. KIH performed data analysis and methodology. SR and SRS conducted lab experiment. KIH and MRI performed the statistical analysis. TRJ performed review and editing. MAI was involved in draft review and editing, methodology, project administration, and investigation. All authors contributed to the article and approved the submitted version.

## Conflict of Interest

The authors declare that the research was conducted in the absence of any commercial or financial relationships that could be construed as a potential conflict of interest.

## Publisher’s Note

All claims expressed in this article are solely those of the authors and do not necessarily represent those of their affiliated organizations, or those of the publisher, the editors and the reviewers. Any product that may be evaluated in this article, or claim that may be made by its manufacturer, is not guaranteed or endorsed by the publisher.

## Footnotes

1
https://www.bioinformatics.babraham.ac.uk/projects/fastqc/

2
https://github.com/katholt/srst2
